# Supplementary material for: Resistome, Mobilome and Virulome Analysis of Shewanella algae and Vibrio spp. Strains Isolated in Italian Aquaculture Centers
Source: Microorganisms. 2020 Apr 15;8(4):572. doi: 10.3390/microorganisms8040572 (PMC7232470; doi:10.3390/microorganisms8040572)
Supplement: Supplementary file 1 [file microorganisms-08-00572-s001.pdf]

SUPPLEMENTARY MATERIAL

Table S1. Summary of genome sizes, %GC content, total number of reads, contigs, predicted proteins, hypothetical proteins and RNAs for each isolate under study.

STRAIN	16S rRNA GENE IDENTIFICATION	GENOME SIZE (bp)	%GC	TOTAL READ	TOTAL CONTIG	PREDICTED PROTEIN	HYPOTHETICAL PROTEIN	RNA ENCODING GENE
**219VB**	*Shewanella algae*	4821382	53.08	8255755	40	3900	782	106
**353M**	*Shewanella algae*	4918582	52.90	7184343	52	3987	810	99
**146bCP**	*Shewanella algae*	4897215	53.03	8131593	57	3991	818	105
**144bCP**	*Shewanella algae*	4893423	53.02	8117126	52	3991	818	112
**57CP**	*Shewanella algae*	4822400	53.08	7527876	67	3867	738	114
**82CP**	*Shewanella algae*	4859761	52.96	8711104	67	3907	771	106
**38LV**	*Shewanella algae*	4782393	53.10	8333193	46	3846	738	106
**83CP**	*Shewanella algae*	4899381	53.00	8713510	55	3918	768	106
**178CP**	*Shewanella algae*	4895527	53.02	8758503	56	3991	817	109
**28AD**	*Vibrio anguillarum*	3868695	44.55	12908870	86	3180	365	99
**60CP**	*Shewanella algae*	4799216	53.07	13809993	72	3853	741	86
**VPE116**	*Vibrio parahaemolyticus*	5089623	45.45	13957982	63	4275	582	91

Table S2. List of heavy metal and other antimicrobial compound resistance genes found in *S. algae* and *Vibrio* spp. genomes.

Compound	Putative gene	Function	Strain
**Arsenic**	*arsR*	Arsenic resistance transcriptional regulator	All
	arsA	Arsenical pump-driving ATPase	All
	arsC	Arsenate reductase	All
	acr3	Arsenical resistance protein *acr3*	All
**Copper**	*cusA*	Cation efflux system protein *cusA*	All
	cusB	Cation efflux system protein *CusB*	All
	copA	Copper-exporting P-type ATPase A	All
	copR	Transcriptional activator protein CopR	All
**Cobalt-zinc-cadmium**	czcA	Cobalt-zinc-cadmium resistance protein CzcA	All *S. algae*, *V. parahaemolyticus* VPE116
	czcB	Cobalt-zinc-cadmium resistance protein CzcB	All *S. algae*, *V. parahaemolyticus* VPE116
	czcC	Cobalt-zinc-cadmium resistance protein CzcC	All *S. algae*, *V. anguillarum* 28AD
	czcD	Cobalt-zinc-cadmium resistance protein CzcD	All
	czcR	Transcriptional activator protein CzcR	All *Vibrio* spp.
	zur	Zinc uptake regulation protein Zur	All *Vibrio* spp.
	znuA	High-affinity zinc uptake system protein ZnuA	All *Vibrio* spp.
	znuB	High-affinity zinc uptake system membrane protein ZnuB	All *Vibrio* spp.
	znuC	Zinc import ATP-binding protein ZnuC	All *Vibrio* spp.
**Chromium**	*chrA*	Chromate transport protein	All *S. algae*
	srpC	Putative chromate transport protein	All *Vibrio* spp.
	chrR	Chromate reductase	All *Vibrio* spp.
**Molybdenum**	*moeA*	Molybdopterin molybdenum transferase	All
	moeB	Molybdopterin-synthase adenylyltransferase	All
**Magnesium**	*corA*	Magnesium transport protein CorA	All *S. algae*
	corC	Magnesium and cobalt efflux protein CorC	All
	mgtE	Magnesium transporter MgtE	All
**Nickel**	*nikR*	Putative nickel-responsive regulator	All *S. algae*
**Bacteriocin**	*lodA*	L-lysine 6-oxidase	All *S. algae*
	lodB	Putative FAD-dependent oxidoreductase LodB	All *S. algae*
	cvpA	Colicin V production protein	All *S. algae*
